# Assessment of early goal-directed therapy guideline adherence: Balancing clinical importance and feasibility

**DOI:** 10.1371/journal.pone.0213802

**Published:** 2019-03-15

**Authors:** Thansinee Saetae, Krit Pongpirul, Rujipat Samransamruajkit

**Affiliations:** 1 Department of Pediatrics, Faculty of Medicine, Chulalongkorn University, Bangkok, Thailand; 2 Department of Preventive and Social Medicine, Faculty of Medicine, Chulalongkorn University, Bangkok, Thailand; 3 Department of International Health, Johns Hopkins Bloomberg School of Public Health, Baltimore, MD, United States of America; Cleveland Clinic, UNITED STATES

## Abstract

**Background:**

Assessing adherence to Early goal-directed therapy (EGDT) is challenging and might account for the negative findings and generalisability of the major trials to a real-life setting. This study was aimed (1) to extract key components of pediatric EGDT guidelines potentially becoming adherence criteria; (2) to classify adherence criteria into complete, clinically important, and feasible; and (3) to compare percent adherence to selected guidelines using the three approaches.

**Methods:**

This study started with review of existing evidence to extract key components of pediatric EGDT guidelines. Modified Delphi method was then conducted in two rounds among national experts to identify feasible and/or clinically important criteria. Data from the national prospective multicenter study “Clinical Effectiveness of the Utilization of Bundled Care for Severe Sepsis and Septicemia Children” at King Chulalongkorn Memorial Hospital (KCMH) during 1 June 2012 and 28 February 2014 was used to compare percentage of adherence across the three approaches.

**Results:**

Of 28 components extracted from the review, 10 were identified by the national experts through the Modified Delphi as feasible whereas 8 were identified as clinically important. Thirty-one severe sepsis patients (48.39% male, median age 3.4 years) were reviewed. Sepsis mortality was 9.7%, a significant reduction from 19% and 42% in 2010 and 2007, respectively. Based on the complete adherence criteria, the percent adherence varied from 60.71% to 89.29% (overall mean 76.84%), with lower adherence in the dead than the survived cases (73.81% vs 77.17%; p = 0.55). The percent adherence varied by criteria used: 69.35%, 76.84%, and 84.52% for clinical importance, complete, and feasibility criteria, respectively.

**Conclusion:**

Adherence determination based on selected clinical importance alone might result in an incorrectly estimated clinical benefit of EGDT guidelines, especially in a resource-limited setting. Both clinical importance and feasibility should be integrated into the development of adherence assessment criteria.

## Introduction

Severe sepsis and septic shock are associated with significant morbidity and mortality [1, 2]. Early goal-directed therapy (EGDT)—a strategy for hemodynamic optimization—has been advocated in clinical practice for sepsis management [2–7]. The Surviving Sepsis Campaign was introduced in 2004 and later updated in 2008 and 2012, covering management of adult and pediatric patients with severe sepsis and septic shock [8, 9]. Unfortunately, many recommendations were based on low-quality adult-focused evidence and expert consensus [10]. Nonetheless, the guideline has been underused; for instance, only 7% of the emergency physicians routinely implemented adult EGDT, based on a survey of 30 academic tertiary care hospitals in the US [11]. Barriers to guideline implementation included a critical shortage of nursing staff, problems in obtaining central venous pressure (CVP) monitoring, and challenges in early identification of patients with sepsis [12, 13]. The other factors were the lack of adequate time and staff, physical space in the emergency department (ED), communication with medical specialties, and identifying appropriate patients.

The negative findings of the ProCESS trial [14], the ARISE study [15], as well as the ProMISe study [16] on the effectiveness of EGDT may not fully support the implementation of a standardised protocol for dynamic care process for adult patients. However, it should be noted that protocol-based care is not just a collection of clinically effective components but a series of inter-related interventions. Although assessing adherence to a complex guideline is challenging, these studies included only cases with full adherence to the guideline defined based on some unempirical adherence criteria.

As high-quality studies usually were conducted in a controlled setting with a full resource, the generalisability of the findings to less feasible settings was unquestionably limited. While information on the protocol adherence in the three major trials was presented in the supplementary appendix of the published articles, some significant discrepancies of compliances between both trials were noted but the relevant discussion was limited [14–16]. It would be unfair for institutions in resource-limited areas that could have benefited from ‘partial’ implementation of the guideline if only the findings from the major studies were used. In the real-life setting, both clinical importance and feasibility of the guideline components should be addressed.

Variation of adherence determination across studies exists whereas the three recent trials contained similar components (Table 1). For example, Mikkelsen et al used only ScvO2 measurement and achievement of hemodynamic goals as main outcome variables to identify factors associated with not initiating EGDT in emergency department [17]. Another study by Crowe et al introduced an extensive list of adherence criteria [18] to assess the effect of EGDT on mortality. Regardless of the negative findings from the modified version, the EGDT for sepsis has still been widely accepted.

**Table 1:** Variation of adherence determination in major studies.

Point of assessment	Studies					
	Mikkelsen 2010	Crowe 2010	O’Neill 2012	ProCESS 2014	ARISE 2014	ProMISe 2015
Initial fluid resuscitation			✓	✓	✓	✓
Appropriate intravenous fluid in 6 hours		✓		✓	✓	✓
Central line placement		✓		✓	✓	✓
Central line placement (only at upper extremity)			✓			
CVP monitoring		✓		✓	✓	✓
ScvO2 measurement at ED	✓					
ScvO2 measurement		✓	✓	✓	✓	✓
Arterial line placement		✓	✓			
Antibiotic administration within 1 hour			✓			
Antibiotic administration within 6 hours		✓				
Appropriate vasopressors administration		✓	✓	✓	✓	✓
Appropriate dobutamine administration		✓		✓	✓	✓
Lactate measurement		✓				
Steroids given		✓				
Keep Hb > 10 gm/dL		✓		✓	✓	✓
Blood cultures drawn in ED		✓				
Achievement of hemodynamic goals at ED	✓					
The use of a standardized order set			✓			

Implementation feasibility of each guideline component was first addressed by O’Neill et al [19]. Seven core components of adult EGDT guideline were assessed for implementation difficulty in a community-based setting. Using percent adherence as the measure, arterial line placement, ScvO2 measurement were the most difficult elements.

Consequently, it was not uncommon to see a “simplified” or “reduced” version of EGDT be implemented in many resource-limited institutions whereas evidence on whether such approach offered comparable outcomes has been limited, especially in pediatric patients. Finding from a prospective cohort study by Sankar et al suggested that even intermittent (rather than continuous) ScvO2 monitoring could still reduce the mortality rate and improve organ dysfunction [20], which was later supported by the recent meta-analysis [21].

It could be assumed that ScvO2 monitoring was one of the critical EGDT components that could be modified when feasibility is of concern. However, whether this concept can be applied to the other EGDT components is still unclear. Empirical evidence on assessing the balance between clinical importance and feasibility of each EGDT component is essential. We synthesized adherence criteria from the pediatric EGDT sepsis guidelines, determined the feasibility of each guideline components, and comparatively assessed the guideline adherence of the care provided to the patients at our institution.

## Materials and methods

### Synthesis of adherence criteria

A panel of five pediatric critical care experts was asked to discuss the EGDT guideline of the Surviving Sepsis Campaign 2012 [9] and selected literature on guideline compliance [14–19]. Finally, a list of key components was produced. EGDT components that were conceptual and/or can be implemented at any time point were classified as ‘general’ whereas the other components were ‘sequential’ components.

### Feasibility determination

A modified Delphi technique was used to determine the feasibility of each guideline component. Feasibility was defined as the likelihood of actual implementation perceived by the clinicians of a specific setting. This can be affected by organizational factors such as the availability of essential equipment and healthcare professional factors such as inadequate skill for central line insertion.

Then, two rounds of the survey were conducted among 20 pediatric pulmonary & critical care specialists. They were regarded as clinical experts in the treatment of severe sepsis and septic shock in children and therefore should be able to identify the most feasible components of the EGDT sepsis guideline. Their responses in the first round were analysed in order to come up with an anonymous summary, which was presented in the second round of the survey.

The questionnaire contained the 28 items identified by the experts as described above. Each respondent was asked to respond to the question “Do you agree that each following items comply in your center?,” using 5-point Likert scale (1 strongly disagree; 2 disagree; 3 neither agree or disagree; 4 agree; 5 strongly agree).

### Exploratory EGDT adherence assessment at an institution

We defined the ten most agreeable components as feasible and all eight components of ProCESS, ARISE, and ProMISe studies as clinically important. Medical records of 31 randomly selected cases (one case per week for every other week) of severe sepsis and septic shock children at King Chulalongkorn Memorial Hospital (KCMH) during 1 June 2012 to 28 February 2014 were reviewed to assess the adherence to EGDT guideline based on the complete, feasibility, and clinical importance criteria.

This study was part of the “Clinical effectiveness of the utilization of bundled care for severe sepsis and septic shock in children at King Chulalongkorn Memorial Hospital” project, which was approved by Journal Research Ethics Committes (JREC), Bangkok, Thailand (JREC 015/55).

## Results

### Synthesis of adherence criteria

The 28 components were divided into 18 general and 10 sequential components (Table 2). All the extracted components from EGDT guideline were listed with the agreement score (mean +/- SD) in the modified Delphi rounds in S1 Table. The changes of means across the two rounds of Modified Delphi technique reflected the extent to which their respondent groups modified their responses to each of the factors whereas final interpretation was made using in the second round.

**Table 2:** Key components of early goal directed therapy.

General Components	Sequential Components
G1. Strategy for patient with ARDS is low tidal volume/optimum PEEP	S1. Choice of initial fluid resuscitation is 0.9%NSS
G2. Appropriate position of central venous catheter is upper > lower	S2. Fluid bolus 20 mL/kg/dose
G3. Indication for FFP transfusion are DIC, TTP, TMA	S3. Maximum bolus fluid is 60 mL/kg
G4. Platelet transfusion when Plt< 10000 or Plt< 20000 + risk bleeding or Plt< 50000 + procedure/surgery or active bleeding	S4. Inotropic drug should be started since 2^nd^ bolus doses of initial fluid resuscitation
G5. Keep Hb ≥ 10 g/dL	S5. 1^st^ inotrope is Dopamine
G6. Initial fluid resuscitation is crystalloid	S6. Maximum dose of Dopamine is 10 mcg/kg/min
G7. Keep urine output > 1 mL/kg/hr	S7. Broad spectrum antibiotics given in 1 hour
G8. Keep ScvO2 > 70%	S8. Hydrocortisone was given in catecholamine resistance shock, suspected or compatible with adrenal insufficiency
G9. Titrate inotrope, keep MAP > 65 mmHg or for maintenance minimum perfusion pressure	S9. Central venous catheter insertion within 1 hour
G10. Central venous pressure monitoring	S10. Follow up lactate and ScvO2 6 hr after treatment
G11. Indication for Dobutamine is normal BP, low cardiac output and high SVR	
G12. Arterial line should be done in cases with inotropic drug used	
G13. Lactate should be < 4 mmol/L	
G14. Lactate should be evaluated	
G15. ScvO2 should be evaluated	
G16. Renal replacement therapy should be done when indicated	
G17. ECMO should be done when refractory septic shock or refractory respiratory failure associated with sepsis	
G18. Immunoglobulin was indicated in severe sepsis and septic shock	

### Feasibility determination

The top ten most feasible components were choices of initial fluid resuscitation (S1); the dose of fluid bolus (S2); the timing of broad-spectrum antibiotics given (S7); strategy for patients with ARDS (G1); hydrocortisone use (S8); crystalloid use as initial fluid resuscitation (G6); site of the central venous catheter (G2); fresh frozen plasma (G3), platelet (G4), and red blood cell transfusion (G5). The ten least feasible components included lactate and ScVO2 level at 6 hours after treatment (S10); maximum dose of fluid bolus (S6); keeping lactate level < 4 mmol/L (G13); lactate evaluation (G14); ScVO2 measurement (G15); renal replacement therapy when indicated (G16); ECMO when indicated (G17); starting inotrope since 2^nd^ dose of fluid resuscitation (S4); IVIg usage (G18); maximum dose of dopamine (S6).

### Exploratory EGDT adherence assessment at an institution

There were 15 male and 16 female patients with median age of 3.4 years (Interquartile Range: 0.7 to 11.8). Twenty-five patients survived, resulting in 9.7% mortality.

Adherence to all 28 items in each case at our institution was shown in S2 Table. For each of the patient, the adhered components were shaded. Percent adherence was calculated by dividing the number of shaded cells by the number of total cells. The row was sorted by percent adherence, from lowest to highest, with the three dead patients listed at the top. None of the patients had perfect adherence. The percent adherence varied from 60.71% to 89.29% (overall mean 76.84%), with insignificantly lower adherence in the dead than the survived cases (73.81% vs 77.17%; p = 0.5514).

**Fig 1:**
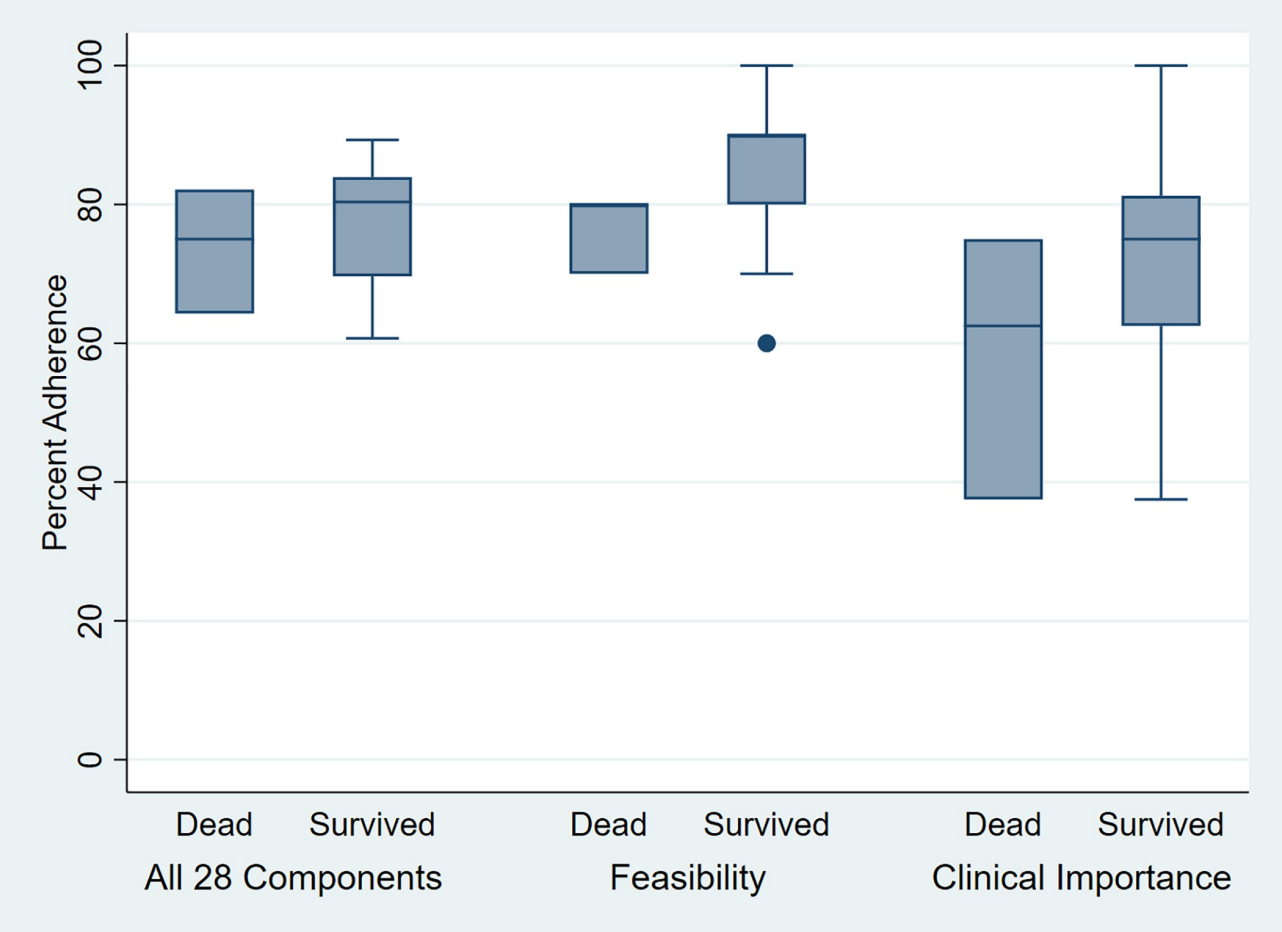
Percent Adherence across various criteria, comparing between dead and survived cases.

Ten perfectly adhered (100%) components included the choice of initial fluid resuscitation (S1); maximum dose of fluid bolus (S3); hydrocortisone use (S8); strategy for the patient with ARDS (G1); platelet transfusion (G4); red blood cell transfusion (G5); dobutamine use (G11); renal replacement when indicated (G16); ECMO when indicated (G17); and immunoglobulin use (G18). The five least adhered components were central venous catheter insertion within one hour (G9; 26%); timing of inotropic drug (S4; 29%); position of central venous catheter (G2; 39%); arterial line insertion (G12; 45%); lactate should be < 4 mmol/L (G13; 52%); lactate evaluation (G14; 52%). Significantly more general components adhered than sequential components (81.8% vs 68.1%). The percent adherence varied by criteria used: 69.35%, 76.84%, and 84.52% for clinical importance, complete, and feasibility criteria, respectively. Adherence seemed to be insignificantly lower among the dead than the survived cases across all criteria (Table 3, Fig 1). The detailed analysis of adherence based on the feasibility and clinical important criteria were presented in S3 and S4 Tables.

**Table 3:** Percent adherence across different criteria.

Percent Adherence	Criteria		
	Complete	Feasibility	Clinical Importance
Number of Criteria	28	10	8
Overall adherence (%; 95%CI)	76.84 (73.51–80.17)	84.52 (80.87–88.16)	69.35 (63.11–75.60)
Adherence in dead cases (%; 95%CI)	73.81 (51.48–96.14)	76.67 (62.32–91.01)	58.33 (10.90–100.06)
Adherence in survived cases (%;95%CI)	77.17 (73.61–80.73)	85.36 (81.48–89.23)	70.54 (64.04–77.03)
	p = 0.5514	p = 0.1535	p = 0.2446

## Discussion

To our knowledge, our work is the first attempt that used a systematic approach to explore details of complex guideline for adherence in children with sepsis. The panel of experts helped to classify the 28 key components, extracted from the literature review, into feasible and clinically important ones. Given the non-representativeness of our experts, these components were used only for the exploration of adherence assessment in this study. Percent adherence assessed by various criteria was calculated, based on the data from a tertiary hospital in Thailand. Findings from our study suggested potential impact of criteria on the assessment of EGDT adherence. Also, even partial adherence to EGDT guideline might still contribute to better outcomes, regardless of the criteria used.

We explored the feasibility of many more EGDT components than the previous study by O’Neill et al (28 vs 7 components). In their study, arterial line insertion, CVP measurement, and ScvO2 monitoring were the least adhered components (42%, 27%, and 15% adherence, respectively [19]. Our study revealed similar findings (45% adherence to arterial line insertion) whereas higher 84% adherence to CVP measurement and ScvO2 monitoring at our facility might reflect the effect of setting (ED vs ICU).

Despite negative effect of EGDT on mortality in the major trials [14–16], a significant reduction of mortality at our site from 42% in 2007 to 19% in 2010 (p<0.02) [22] and 9.7% in this study still supported the clinical benefit of EGDT, even when partially implemented. However, many other factors could contribute to the reduction of mortality so the direct clinical benefit of EGST could not be inferred from our data. Based on the adherence criteria used by ProCESS, ARISE, and ProMISe studies, 94% (29/31) of our patients would have been excluded, suggesting that including only fully adhered cases might lead to potentially incorrect estimation of EGDT effect on clinical outcomes in the real-life setting.

The incremental gain from EGDT implementation might be relatively small in fully equipped settings with a high standard of care, as suggested by negative findings from the major trials [14–16]. One limitation of these well-designed clinical trials was the exclusion of partially adhered cases. However, findings from our study suggested that a partially implemented EGDT might still improve clinical outcomes, at least in a developing country like Thailand.

To deal with imperfect adherence in real life setting, a balanced determination between clinically important and feasible components must be carefully considered using prioritization approaches presented in Table 4. In the ideal setting, all the 28 components must be fully implemented. Otherwise, the two clinically important and feasible components (G5 and S2) must be done. Next, barriers to implementing the other six clinically important components (G8, G9, G11, G15, S9, S10) should be removed. When all clinically important components are fully adhered, the eight feasible-yet-less important components (G1, G2, G3, G4, G6, S1, S7, S8) are encouraged. As feasibility is context-specific, each institution must identify feasible components in order to come up with the prioritized opportunity for improvement of EGDT adherence.

**Table 4:** Prioritized opportunity for improvement of EGDT adherence.

		Clinical Importance Criteria	
		Yes	No
Feasibility Criteria	Yes	• Keep Hb ≥ 10 g/dL (G5) • Fluid bolus 20 mL/kg/dose (S2)	• Strategy for patient with ARDS is low tidal volume/optimum PEEP (G1) • Appropriate position of central venous catheter is upper > lower (G2) • Indication for FFP transfusion are DIC, TTP, TMA (G3) • Platelet transfusion when Plt < 10000 or Plt < 20000 + risk bleeding or Plt < 50000 + procedure/surgery or active bleeding (G4) • Initial fluid resuscitation is crystalloid (G6) • Choice of initial fluid resuscitation is 0.9%NSS (S1) • Broad spectrum antibiotics given in 1 hour (S7) • Hydrocortisone was given in catecholamine resistance shock, suspected or compatible with adrenal insufficiency (S8)
	No	• Keep ScvO2 > 70% (G8) • Titrate inotrope, keep MAP > 65 mmHg or for maintenance minimum perfusion pressure (G9) • Indication for Dobutamine is normal BP, low cardiac output and high SVR (G11) • ScvO2 should be evaluated all cases (G15) • Central venous catheter insertion within 1 hour (S9) • Follow up lactate and ScvO2 6 hr after treatment (S10)	• Keep urine output > 1 mL/kg/hr (G7) • Central venous pressure monitoring (G10) • Arterial line should be done in cases with inotropic drug used (G12) • Lactate should be < 4 mmol/L (G13) • Lactate should be evaluated (G14) • Renal replacement therapy should be done when indicated (G16) • ECMO should be done when refractory septic shock or refractory respiratory failure associated with sepsis (G17) • Immunoglobulin was indicated in severe sepsis and septic shock (G18) • Maximum bolus fluid is 60 mL/kg (S3) • Inotropic drug should be started since 2nd bolus doses of initial fluid resuscitation (S4) • 1st inotrope is Dopamine (S5) • Maximum dose of Dopamine is 10 mcg/kg/min (S6)

## Conclusions

Adherence determination based on selected clinical importance alone might result in an incorrectly estimated benefit of clinical guideline, especially in a resource-limited setting. This pitfall of measurement may confound the effect of EGDT sepsis guideline on clinical outcomes. Both clinical importance and feasibility should be integrated into the development of adherence assessment criteria.

## Supporting information

S1 Table Two-Round modified Delphi on feasibility of EGDT components. (DOCX)

S2 Table Adherence to EGDT components at King Chulalongkorn Memorial Hospital. (DOCX)

S3 Table Adherence, using feasibility criteria in King Chulalongkorn Memorial Hospital. (DOCX)

S4 Table Adherence, using Clinical Importance criteria in King Chulalongkorn Memorial Hospital. (DOCX)

S1 File King Chulalongkorn Memorial Hospital EGDT compliance data. (XLS)

## Abbreviations

ARISE: Australasian Resuscitation in Sepsis Evaluation
ARDS: Acute Respiratory Distress Syndrome
CVP: Central Venous Pressure
ECMO: Extracorporeal Membrane Oxygenation
EGDT: Early Goal-Directed Therapy
ED: Emergency Department
ICU: Intensive Care Unit
IVIg: Intravenous Immunoglobulin
KCMH: King Chulalongkorn Memorial Hospital
ProCESS: Protocolized Care for Early Septic Shock
ProMISe: Protocolised Management in Sepsis
ScvO2: Central Venous Oxygen Saturation
SD: Standard Deviation
