# Supplementary material for: Assessment of early goal-directed therapy guideline adherence: Balancing clinical importance and feasibility
Source: PLoS One. 2019 Mar 15;14(3):e0213802. doi: 10.1371/journal.pone.0213802 (PMC6420253; doi:10.1371/journal.pone.0213802)
Supplement: S1 Table — (DOCX) [file pone.0213802.s001.docx]

S1 Table. Two-Round Modified Delphi on Feasibility of EGDT Components

| Components | Round 1 | | Round 2 | |
| --- | --- | --- | --- | --- |
|  | Mean | SD | Mean | SD |
| Choice of initial fluid resuscitation is 0.9%NSS | 4.71 | 0.56 | 5.00 | 0.00 |
| Fluid bolus 20 mL/kg/dose | 4.81 | 0.40 | 4.95 | 0.21 |
| Broad spectrum antibiotics given in 1 hour | 4.95 | 0.22 | 4.91 | 0.43 |
| Strategy for patient with ARDS is low tidal volume/optimum PEEP | 4.95 | 0.22 | 4.91 | 0.29 |
| Hydrocortisone was given in catecholamine resistance shock, suspected or compatible with adrenal insufficiency | 4.62 | 0.59 | 4.82 | 0.50 |
| Initial fluid resuscitation is crystalloid | 4.52 | 1.03 | 4.77 | 0.87 |
| Appropriate position of central venous catheter is upper > lower | 4.48 | 0.81 | 4.77 | 0.43 |
| Indication for FFP transfusion are DIC, TTP, TMA | 4.67 | 0.58 | 4.77 | 0.53 |
| Platelet transfusion when Plt < 10000 or Plt < 20000 + risk bleeding or Plt < 50000 + procedure/surgery or active bleeding | 4.67 | 0.58 | 4.77 | 0.53 |
| Keep Hb ≥ 10 g/dL | 4.57 | 0.60 | 4.68 | 0.65 |
| Central venous catheter insertion within 1 hour | 4.43 | 0.75 | 4.64 | 0.58 |
| Keep urine output > 1 mL/kg/hr | 4.57 | 0.68 | 4.64 | 0.66 |
| Keep ScvO2 > 70% | 4.52 | 0.6 | 4.59 | 0.73 |
| Titrate inotrope, keep MAP > 65 mmHg or for maintenance minimum perfusion pressure | 4.62 | 0.50 | 4.55 | 0.67 |
| 1^st^ inotrope is Dopamine | 4.19 | 0.87 | 4.41 | 0.73 |
| Central venous pressure monitoring | 4.33 | 0.58 | 4.41 | 0.67 |
| Indication for Dobutamine is normal BP, low cardiac output and high SVR | 4.05 | 0.92 | 4.32 | 0.57 |
| Arterial line should be done in cases with inotropic drug used | 4.24 | 0.89 | 4.32 | 0.72 |
| Follow up lactate and ScvO2 6 hr after treatment | 4.14 | 0.85 | 4.27 | 0.94 |
| Maximum bolus fluid is 60 mL/kg | 4.14 | 1.15 | 4.23 | 1.02 |
| Lactate should be < 4 mmol/L | 4.29 | 0.91 | 4.23 | 0.69 |
| Lactate should be evaluated all cases | 4.33 | 0.78 | 4.18 | 0.91 |
| ScvO2 should be evaluated all cases | 3.95 | 0.97 | 4.09 | 0.87 |
| Renal replacement therapy should be done when indicated | 3.90 | 1.00 | 4.05 | 1.05 |
| ECMO should be done when refractory septic shock or refractory respiratory failure associated with sepsis | 4.00 | 0.95 | 4.00 | 0.98 |
| Inotropic drug should be started since 2^nd^ bolus doses of initial fluid resuscitation | 3.81 | 0.98 | 3.95 | 1.00 |
| Immunoglobulin was indicated in severe sepsis and septic shock | 3.76 | 1.09 | 3.59 | 1.10 |
| Maximum dose of Dopamine is 10 mcg/kg/min | 3.05 | 1.53 | 3.09 | 1.15 |
