# Supplementary material for: Assessment of early goal-directed therapy guideline adherence: Balancing clinical importance and feasibility
Source: PLoS One. 2019 Mar 15;14(3):e0213802. doi: 10.1371/journal.pone.0213802 (PMC6420253; doi:10.1371/journal.pone.0213802)
Supplement: S3 Table — (DOCX) [file pone.0213802.s003.docx]

| Gender | Age  (years) | General Components | | | | | | Sequential Components | | | | Adherence |
| --- | --- | --- | --- | --- | --- | --- | --- | --- | --- | --- | --- | --- |
|  |  | G1 | G2 | G3 | G4 | G5 | G6 | S1 | S2 | S7 | S8 |  |
| Female | 13.0 |  |  |  |  |  |  |  |  |  |  | 70% |
| Female | 16.0 |  |  |  |  |  |  |  |  |  |  | 80% |
| Female | 1.6 |  |  |  |  |  |  |  |  |  |  | 80% |
| Female | 14.1 |  |  |  |  |  |  |  |  |  |  | 60% |
| Male | 0.3 |  |  |  |  |  |  |  |  |  |  | 70% |
| Female | 4.9 |  |  |  |  |  |  |  |  |  |  | 70% |
| Male | 4.3 |  |  |  |  |  |  |  |  |  |  | 70% |
| Male | 0.2 |  |  |  |  |  |  |  |  |  |  | 80% |
| Female | 1.2 |  |  |  |  |  |  |  |  |  |  | 80% |
| Male | 3.7 |  |  |  |  |  |  |  |  |  |  | 80% |
| Female | 13.2 |  |  |  |  |  |  |  |  |  |  | 80% |
| Male | 11.8 |  |  |  |  |  |  |  |  |  |  | 80% |
| Female | 9.3 |  |  |  |  |  |  |  |  |  |  | 80% |
| Female | 0.9 |  |  |  |  |  |  |  |  |  |  | 80% |
| Female | 1.1 |  |  |  |  |  |  |  |  |  |  | 80% |
| Male | 13.0 |  |  |  |  |  |  |  |  |  |  | 90% |
| Male | 0.4 |  |  |  |  |  |  |  |  |  |  | 90% |
| Female | 4.6 |  |  |  |  |  |  |  |  |  |  | 90% |
| Female | 15.3 |  |  |  |  |  |  |  |  |  |  | 90% |
| Male | 9.8 |  |  |  |  |  |  |  |  |  |  | 90% |
| Female | 0.4 |  |  |  |  |  |  |  |  |  |  | 90% |
| Male | 0.7 |  |  |  |  |  |  |  |  |  |  | 90% |
| Male | 0.2 |  |  |  |  |  |  |  |  |  |  | 90% |
| Female | 0.3 |  |  |  |  |  |  |  |  |  |  | 90% |
| Female | 13.3 |  |  |  |  |  |  |  |  |  |  | 90% |
| Male | 1.7 |  |  |  |  |  |  |  |  |  |  | 90% |
| Male | 4.1 |  |  |  |  |  |  |  |  |  |  | 90% |
| Male | 1.7 |  |  |  |  |  |  |  |  |  |  | 100% |
| Male | 5.9 |  |  |  |  |  |  |  |  |  |  | 100% |
| Male | 0.2 |  |  |  |  |  |  |  |  |  |  | 100% |
| Female | 0.7 |  |  |  |  |  |  |  |  |  |  | 100% |
|  |  | 100% | 39% | 97% | 100% | 100% | 90% | 100% | 58% | 61% | 100% | 84% |

S3 Table. Adherence, using Feasibility Criteria in King Chulalongkorn Memorial Hospital
